# Synthesis of a derivative of α-D-Glc*p*(1->2)-D-Gal*f* suitable for further glycosylation and of α-D-Glc*p*(1->2)-D-Gal, a disaccharide fragment obtained from varianose

**DOI:** 10.3762/bjoc.8.241

**Published:** 2012-12-07

**Authors:** Carla Marino, Carlos Lima, Karina Mariño, Rosa M de Lederkremer

**Affiliations:** 1 CIHIDECAR-CONICET-UBA, Departamento de Química Orgánica, Facultad de Ciencias Exactas y Naturales, UBA, Buenos Aires (1428), Argentina; 2 Laboratorio de Glicómica Funcional y Molecular, Instituto de Biología y Medicina Experimental (IBYME), CONICET, Buenos Aires (1428), Argentina

**Keywords:** α-D-galactofuranose, glucosylgalactofuranose, glycosylaldonolactone, *Penicillium varians*, varianose

## Abstract

The presence of galactofuranoyl units in infectious microorganisms has prompted the study of the metabolic pathways involved in their incorporation in glycans. Although much progress has been made with respect to the biosynthesis of β-D-Gal*f*-containing glycoconjugates, the mechanisms by which α-D-Gal*f* units are incorporated remain unclear. *Penicillium varians* is a non-pathogenic fungus that produces varianose, a polysaccharide containing both α- and β-D-Gal*f* units, which can be used as a model for biosynthetic studies on α-D-Gal*f* incorporation. Synthetic oligosaccharide fragments related to varianose are useful as potential substrates or standards for characterization of the α-galactofuranosyl transferases. In this paper we report a straightforward procedure for the synthesis of α-D-Glc*p*(1→2)-D-Gal (**1**) and the use of this compound to monitor the natural disaccharide released from varianose by mild acid degradation. The synthesis, performed by the glycosylaldonolactone approach, involved a glucosylgalactofuranose derivative, suitable for the synthesis of higher oligosaccharides with an internal D-Gal*f.*

## Introduction

Carbohydrates are involved in a wide range of biological processes; they play important roles in host–pathogen interactions and in the immune response, where protein–glycan interactions mediate a variety of biological processes, including cell trafficking, activation, differentiation and survival [1–2]. The variety and distinctive chemical properties of carbohydrates make them ideal units of condensed information, as variability is achieved not only in the glycan sequence but also in their spatial distribution. As a result, establishing the connection between the glycan structure and its biological function, a discipline called functional glycomics, is one of the most challenging areas nowadays and requires a broad interdisciplinary approach.

**Figure 1:**
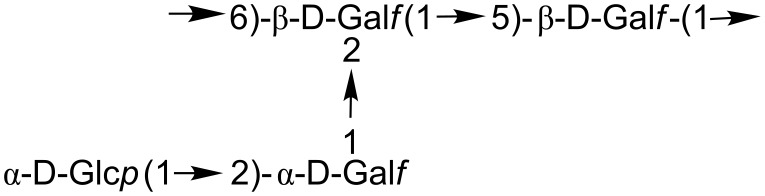
Repeating unit of varianose.

*Penicillium varians* elaborates an unusual polysaccharide called varianose [3] that contains galactofuranose (Gal*f*) in both α and β-configurations in the repeating unit (Figure 1). Further studies established that the galactofuran of alternating β(1→6) and β(1→5)Gal*f* units is branched at the O-2 of the (1→5)-linked Gal*f* with the disaccharide α-D-Glc*p*(1→2)-α-D-Gal*f* [4]. Mild acid hydrolysis, which preferentially cleaves the furanosidic linkages, allowed the isolation and characterization of the disaccharide with the reducing galactose isomerized to the more stable pyranose form. The same disaccharide unit is present in the cell-wall polysaccharide of *Talaromyces flavus* [5] and in the extracellular polysaccharide of *Penicillium vermiculatum* (anamorph of *T. flavus*) [6].

The biosynthesis of Gal*f* has been studied in several laboratories in the past few years, and reviews on the subject have been published [7–8]. The main interest lies in the presence of Gal*f* in infectious microorganisms from bacteria to protozoa and in the fact that, being absent in mammals, the metabolism of Gal*f* is a good target for chemotherapy [9–11]. In particular, the unique UDP-galactopyranose mutase necessary for the production of the Gal*f* nucleotide has been thoroughly studied and characterized in several microorganisms producing β-galactofurans [7–8,10]. However, the enzymes involved in the metabolic incorporation of α-D-Gal*f* were not studied. If UDP-α-D-Gal*f* is also the donor for the incorporation of α-Gal*f*, the mechanism should be different since the stereochemistry of the β-galactofuranosidic linkage in the products indicates an inverting mechanism.

Also lately, galactofuranosyl transferases (GalfT) catalyzing β-Gal*f* incorporation in glycans have been identified. Two bifunctional GalfTs are required for galactan biosynthesis in *Mycobacterium tuberculosis*, GalfT1 and GalfT2, able to construct both, β-Gal*f*(1→5)Gal*f* and β-Gal*f*(1→6)Gal*f* [12–14]. Synthetic oligosaccharides have been used to characterize these GalfTs and it was demonstrated that a single active site in GalfT2 is responsible for both activities [15]. The crystal structures of both, free and UDP-bound GalfT2 have been recently determined [16]. Despite all this progress, no Gal*f* transferases (GalfTα) responsible for the transfer of α-Gal*f* to a glycan acceptor have been described. On the other hand, radiolabeled substrates for studies on both galactofuranosidases have been prepared [17]. Varianose, which is produced by a non-pathogenic fungus, is a good model for studies on the GlfTα. The constitutive oligosaccharides could be used as substrates or as standards for the identification of the products. A pentasaccharide fragment containing both, α and β-galactofuranosyl residues was synthesized by Bay and Lowary [18]. The two possible trisaccharide units of β-Gal*f* in the backbone linked 1→5 and 1→6, synthesized in our laboratory and by other groups [19–20], are good substrates for studying the branching by the disaccharide. This may occur by sequential incorporation of Gal*f* and then Glc*p*, or of the preformed disaccharide α-D-Glc*p*(1→2)-D-Gal (**1**). In the present paper we describe a facile chemical synthesis of disaccharide **1** and its use as a synthetic standard for the identification of the natural disaccharide obtained from varianose, by means of chromatographic techniques. The synthesis of a Glc*p*(1→2)-α-D-Gal*f* derivative, compound **8**, useful for the preparation of higher oligosaccharides with an internal Gal*f*, is also described. The strategy relies on glucosylation of a convenient derivative of D-galactono-1,4-lactone as a precursor of D-Gal*f* [10].

## Results and Discussion

### Synthesis of 2-*O*-(per-*O*-benzyl-α-D-Glc*p*)-per-*O*-benzoyl-α,β-D-Gal*f* (**8**) and α-D-Glc*p* (1→2)-D-Gal (**1**)

**Scheme 1:**
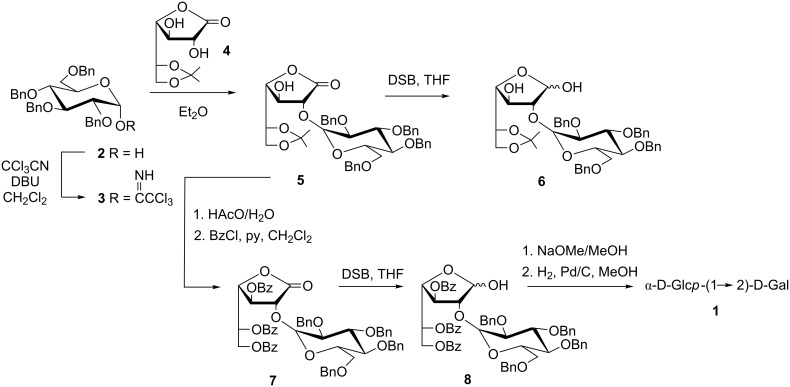
Synthesis of key intermediate α-2-*O*-(2,3,4,6-tetra-*O*-benzyl-α-D-Glc*p*)-3,5,6-tri-*O*-benzoyl-α,β-D-Gal*f* (**8**) and α-D-Glc*p* (1→2)-D-Gal (**1**).

D-Galactono-1,4-lactone has long been used in our laboratory as a good precursor of the galactofuranosyl units in disaccharides, by using the glycosylaldonolactone approach. This method has the advantage of providing selectively substituted derivatives of the lactone, due to the differential reactivity of the HO groups [21–22]. These derivatives can be used as acceptors, with the anomeric center virtually masked as a carbonyl group, which may be later selectively reduced to the hemiacetal with diisoamylborane (DSB). By this strategy the synthesis of many oligosaccharide constituents of pathogenic microorganisms has been achieved [10]. The isopropylidene derivative **4** [23] has been regioselectively glycosylated at HO-2 by the galactofuranosyl iodide method, affording the β-(1→2) linkage with complete regioselectivity [24]. Derivative **4** was also used for the synthesis of α-D-Gal*f*-(1→2)-D-galactose by the same glycosylaldonolactone approach [25]. The same derivative **4** was envisaged as precursor of **1** due to the high regioselectivity previously achieved in glycosylation at HO-2. Additionally, this procedure would afford derivative **8**, a valuable intermediate for the synthesis of larger varianose fragments (Scheme 1).

Starting from tetra-*O*-benzyl-D-Glc*p* (**2**), the trichloroacetimidate **3** was prepared by treatment with trichloroacetonitrile in the presence of 1,8-diazabicyclo[5.4.0]undec-7-ene (DBU) [26–27]. Condensation of **3** with the isopropylidene derivative **4** in Et_2_O, employing TMSOTf as catalyst, proceeded with complete regio- and stereoselectivity, affording the α-glycosylaldonolactone **5** in 79% yield (Scheme 1). The α-anomeric configuration and the regioselectivity of the glycosylation were confirmed on the basis of the NMR spectra. In the ^1^H NMR spectrum the H-1’ signal appeared as a doublet centered at 4.82 ppm with *J*_1,2_ = 3.9 Hz, characteristic of α-D-glucopyranosides. The multiplet centred at 4.32 ppm, assigned to H-3, showed coupling with the HO-3 (δ 4.62, *J*_3,HO_ = 4.3 Hz). On deuteration this signal disappeared and the H-3 signal collapsed into an apparent triplet. On the other hand, in the ^13^C NMR spectrum of **5** the signal corresponding to C-1 appeared at δ 170.1, shifted 4 ppm in comparison with the corresponding signal of **4**, as result of glycosylation at the vicinal position [28].

For the reduction of the lactone function, compound **5** was treated with DSB, as previously reported for other glycosylaldonolactones [10,25,28]. Although lactol **6** was obtained, the reaction was slow and even after several days it was not complete (Scheme 1). Acetylation of the HO-3 of **5** did not improve the reduction step. Certainly, the presence of the α-D-Glc*p* unit in the HO-2 would hinder the proper diisoamylborane complexation. In order to relieve the conformational strain we prepared derivative **7** by deisopropylidenation followed by benzoylation of **5**. Diisoamylborane reduction of **7** was more efficient and afforded **8** in 76% yield after 72 h of reaction and column chromatography purification. It is worth mentioning that starting compound **7** was recovered from faster moving fractions, thus, β-elimination on the lactone unit, or other side reactions, did not occur. The ^1^H NMR spectrum of **8** showed that it was obtained as an anomeric mixture in a 1:1 α/β ratio. The anomeric signals were observed at δ 5.55 (dd, H-1α) and 5.50 (d, H-1β) and after D_2_O exchange they were simplified to a doublet and a broad singlet, respectively. The ^13^C NMR spectrum of **8** showed signals at 97.2 and 96.7 ppm for the glucopyranosyl unit of both anomers and signals at δ 100.4 and 99.3 for C-1 of the β and α-anomers, respectively. Derivative **8** is ready to be activated by the trichloroacetimidate method, for the synthesis of larger oligosaccharides.

Conventional de-*O*-benzoylation and subsequent hydrogenolysis of **8** afforded disaccharide **1** in good yield (Scheme 1). The NMR spectra of **1** showed equilibrium between the disaccharide with the reducing end in the pyranosyl and a lower amount in the furanosyl configuration. Thus, the ^13^C NMR spectrum showed resonances at 97.9 and 96.1 ppm, corresponding to C-1’ and signals at 96.6 and 89.6 ppm, due to C-1 of both anomers of α-D-Glc*p*(1→2)-D-Gal*p*. Less intense signals at δ 104.0 and 100.5 corresponded to the anomeric carbons of the reducing unit in α-D-Glc*p*(1→2)-D-Gal*f*. The ^1^H NMR spectrum showed for α-D-Glc*p*(1→2)-D-Gal*p* doublets at 5.46 (*J* = 3.6 Hz) and 4.73 ppm (*J* = 7.8 Hz), characteristic of α and β-galactopyranosyl units.

Per-*O*-benzylated disaccharide **1** was first synthesized with a low yield by condensation of per-*O*-benzyl-α-D-glucopyranosyl chloride with benzyl 3,4,6-tri-*O*-benzyl-α-D-galactopyranoside. This acceptor was obtained in seven steps from 6-*O*-allyl-1,2:3,4-di-*O*-isopropylidene-α-D-galactopyranose [29]. The synthesis was improved by using the 1-*O*-(*N*-methyl)acetimidyl-2,3,4,6-tetra-*O*-benzyl-β-D-glucopyranose as donor, although it was condensed with the same galactosyl acceptor, which involved a laborious procedure [30]. A general strategy for the synthesis of α-(1→2)-linked disaccharides by a regioselective one-pot protection–glycosylation was later described, including a derivative of **1** [31]. The glycosylaldonolactone approach now described provides a straightforward method for the synthesis of disaccharide **1**.

### Identification of the disaccharide released from varianose with synthetic Glc*p*(1→2)-α-D-Gal (1) by HPAEC–PAD

Varianose was isolated from a culture of *Penicillium varians* [3] and its identity was proved by methylation and GLC–MS analysis. D-Galactose, D-mannose and D-glucose in the ratio 70:15:15 and the corresponding diagnostic peaks for the methylated sugars were observed, as previously described [4] (not shown).

**Figure 2:**
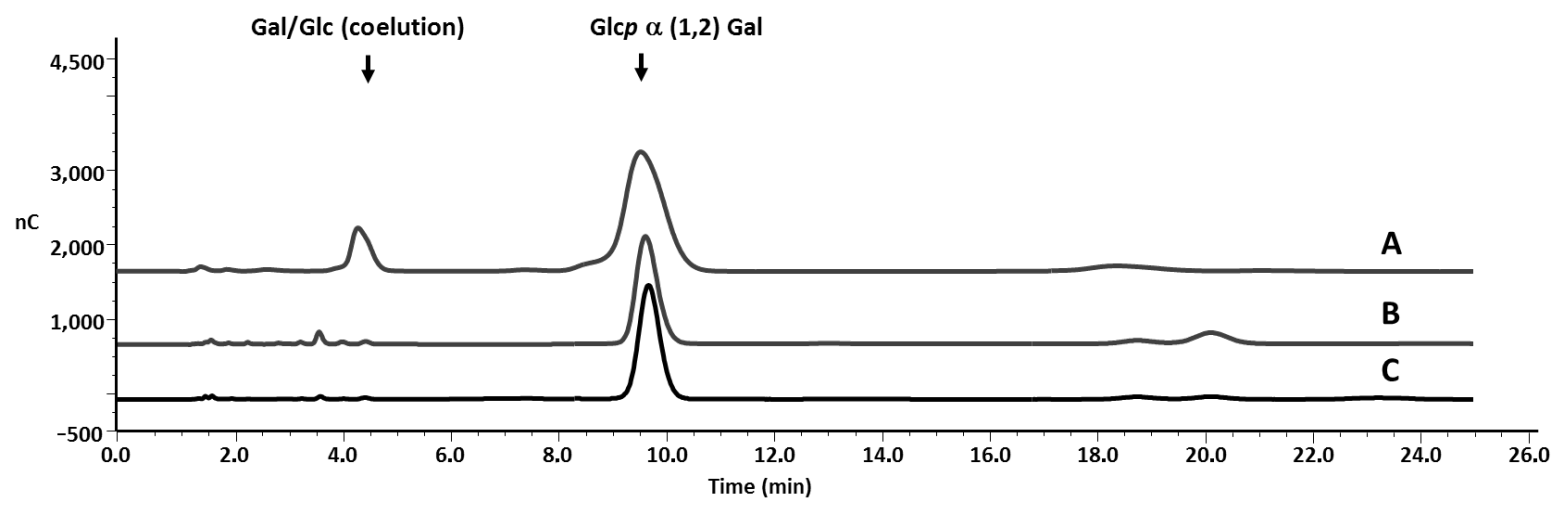
HPAEC-PAD analysis of disaccharide α-D-Glc*p*(1→2)-D-Gal (**1**) obtained by hydrolysis of varianose and by chemical synthesis. The separation was performed on a CarboPac PA-10 column under condition 1, as described in the Experimental section. (A) Varianose isolated from a *P. varians* culture was hydrolyzed with 0.1 M TFA for 90 min at 85 °C. The included fraction of Biogel P-2 contains monosaccharides (*t*_R_ 4.4 min) and disaccharide **1** (*t*_R_ 9.8 min). (B) Synthetic sample of **1**. (C). Co-chromatography of natural and synthetic disaccharide **1**.

With the intention to use Glc*p*(1→2)-α-D-Gal (**1**) for biosynthetic studies on the α-D-Gal*f* incorporation in varianose, we developed a method for the identification of the fragment obtained from the polysaccharide. The mildly acidic degradation conditions selectively cleaved the α-furanosidic linkages of varianose (Figure 1) in accordance with the higher stability of the β-galactofuranosidic linkages [32]. After column chromatography on a Biogel P-2 column, the disaccharide in the included fraction (Figure 2A) could be separated by further chromatography from the monosaccharides (not shown). As depicted in Figure 2, high pH anion exchange chromatography with pulse amperometric detection (HPAEC–PAD) showed that the synthetic disaccharide (Figure 2B) coeluted with the natural product (Figure 2C).

## Conclusion

In conclusion, we have reported a very simple procedure for the synthesis of Glc*p*(1→2)-α-D-Gal (**1**), and its use as a tool for the identification of the disaccharide linked by α-D-Gal*f* to the backbone of β-D-galactofuranosyl residues in the polysaccharide varianose. The synthetic disaccharide is a useful standard to monitor the branching by using HPAEC–PAD. Moreover, the intermediate glucosylgalactofuranosyl derivative **8** could be further glycosylated to obtain larger oligosaccharides from varianose.

## Experimental

### General methods

Analytical thin layer chromatography (TLC) was performed on Silica Gel 60 F_254_ (Merck) aluminum supported plates (layer thickness 0.2 mm) with solvent systems given in the text. Visualization of the spots was effected by exposure to UV light or dipping in a solution of 10% (v/v) sulfuric acid in EtOH, containing 0.5% *p*-anisaldehyde. Column chromatography was carried out with silica gel 60 (230–400 mesh, Merck). Optical rotations were measured with a Perkin-Elmer 343 digital polarimeter. Nuclear magnetic resonance (NMR) spectra were recorded with a Bruker AMX 500 spectrometer. Assignments of ^1^H and ^13^C were assisted by 2D ^1^H COSY and HSQC experiments. High-resolution mass spectra (HRMS–ESI^+^) were recorded on a Bruker micrOTOF-Q II spectrometer.

### Synthesis

**2-*O*-(2,3,4,6-Tetra-*O*-benzyl-α-D-glucopyranosyl)-5,6-*O*-isopropylidene-D-galactono-1,4-lactone (5):** To a stirred solution of 2,3,4,6-tetra-*O*-benzyl-α-D-glucopyranose (1.20 g, 2.2 mmol) and trichloroacetonitrile (3.7 mL, 37.4 mmol) in dry CH_2_Cl_2_ (15 mL), cooled to 0 °C, was slowly added DBU (0.38 mL, 2.6 mmol). After 1 h of stirring, the solution was carefully concentrated under reduced pressure at room temperature, and the residue was purified by short-column chromatography (hexane/EtOAc 4:1) to give 1.28 mg (85%) of the trichloroacetimidate of **3** as a syrup, *R*_f_ 0.58 (hexane/EtOAc 2:1). A stirred suspension of the trichloroacetimidate of **3** (1.28 g, 1.88 mmol), 5,6-*O*-isoprolyliden-D-galactono-1,4-lactone (**4**, 0.48 g, 2.24 mmol) [16] and 4 Å powdered molecular sieves (0.5 g) in anhydrous Et_2_O (12 mL) was cooled to −78 °C, and TMSOTf (0.11 mL, 0.62 mmol) was slowly added. After 48 h of stirring at 5 °C, the mixture was quenched by addition of saturated aq NaHCO_3_ (15 mL) and then extracted with CH_2_Cl_2_. After purification by column chromatography (hexane/EtOAc 4:1) compound **5** was obtained as a syrup (0.98 g, 71%), *R*_f_ 0.28 (hexane/EtOAc 2:1); [α]_D_ +18.1 (*c* 1, CHCl_3_); ^1^H NMR (CDCl_3_, 500 MHz) δ 7.47–7.22 (20 H, aromatic), 5.02 (d, *J* = 10.8 Hz, 1H, C*H*HPh), 4.84 (d, *J* = 10.9 Hz, 1H, C*H*HPh), 4.82 (d, *J* = 3.9 Hz, 1H, H-1’), 4.79 (d, *J* = 10.9 Hz, 1H, C*H*HPh), 4.78 (d, *J* = 10.9 Hz, 1H, C*H*HPh), 4.71 (d, *J* = 11.8 Hz, 1H, C*H*HPh), 4.62 (d, 1H, *J* = 4.3 Hz, HO-3), 4.49 (d, *J* = 11.8 Hz, 1H, C*H*HPh), 4.46 (d, *J* = 11.8 Hz, 1H, C*H*HPh), 4.45 (d, *J* = 10.9 Hz, 1H, C*H*HPh), 4.32 (m, 1H, H-3), 4.18 (m, 1H, H-5), 4.09 (d, *J* = 8.2 Hz, 1H, H-2), 3.99 (m, 1H, H-3’), 3.97 (dd, *J* = 8.5, 6.8 Hz, 1H, H-6a), 3.94 (m, 2H, H-4,5), 3.88 (dd, *J* = 8.5, *J* = 7.2 Hz, 1H, H-6b), 3.68 (dd, *J* = 9.7, *J* = 1.7 Hz, 1H, H-6’a), 3.59 (dd, *J* = 9.5, 3.9 Hz, 1H, H-2’), 3.37 (apparent t, *J* = 9.0 Hz, 1H, H-6’b), 3.27 (dd, *J* = 10.1, 8.8 Hz, 1H, H-4’), 1.42 (s, 3H, (C*H*_3_)_2_C), 1.39 (s, 3H, (C*H*_3_)_2_C); ^13^C NMR (CDCl_3_, 125.8 MHz) δ 170.1 (C-1), 138.5, 138.0, 137.5, 128.5, 128.3 (aromatic), 110.0 (*C*(CH_3_)_2_), 98.1 (C-1’), 82.4 (C-2), 81.4 (C-3’), 79.7 (C-2’), 78.4 (C-4), 77.7 (C-4’), 75.7 (*C*H_2_Ph), 75.1 (*C*H_2_Ph), 74.0 (C-5), 73.6 (C-3), 73.1 (*C*H_2_Ph), 72.9 (*C*H_2_Ph), 70.8 (C-5’), 69.43 (C-6), 64.9 (C-6’), 26.08, 25.68 (C(*C*H_3_)_2_); Anal. calcd for C_43_H_48_O_11_: C, 69.71; H, 6.53; O, 23.76; found: C, 69.74; H, 6.67.

**2-*O*-(2,3,4,6-Tetra-*O*-benzyl-α-D-glucopyranosyl)-3,5,6-tri-*O*-benzoyl-D-galactono-1,4-lactone (7):** A solution of compound **5** (0.50 g, 0.67 mmol) in HOAc/H_2_O 4:1 (5 mL) was heated at 60 °C for 2.5 h. TLC analysis of the syrup showed total conversion of the starting compound (*R*_f_ 0.79, EtOAc/hexane 2:1) into a product of *R*_f_ 0.23. The solution was poured into ice/water and diluted with CH_2_Cl_2_, and the organic layer was washed with NaHCO_3_ (ss) and water, dried (Na_2_SO_4_), and concentrated. The syrup obtained was dried under vacuum, dissolved in dried CH_2_Cl_2_ and cooled to 0 °C. Then, pyridine (1.0 mL) and BzCl (0.8 mL) were added. After 1 h of stirring, TLC analysis showed a single product of R*_f_* 0.84 (EtOAc/hexane 2:1). The solution was poured into ice/water and diluted with CH_2_Cl_2_. The organic layer was washed with NaHCO_3_ (ss) and water, dried (Na_2_SO_4_), and concentrated. After purification by column chromatography (EtOAc/hexane 5:2) syrupy compound **7** (0.47 g, 69%) was obtained, *R*_f_ 0.48 (hexane/EtOAc 2:1), [α]_D_ +8.3 (*c* 2, CHCl_3_); ^1^H NMR (CDCl_3_, 500 MHz) δ 8.08–6.95 (35H, aromatic), 5.98 (m, 1H, H-5), 5.78 (apparent t, *J* = 6.1 Hz, 1H, H-3), 5.57 (d, *J* = 3.6 Hz, 1H, H-1’), 4.95 (d, *J* = 6.5 Hz, 1H, H-2), 4.90 (d, *J* = 11.5 Hz, 1H, C*H*HPh), 4.90 (dd, *J* = 2.9, 5.9 Hz, 1H, H-4), 4.77 (d, *J* = 11.0 Hz, 1H, C*H*HPh), 4.73–4.70 (m, 2H, H6a, H-6b), 4.72 (d, *J* = 11.0 Hz, 1H, C*H*HPh), 4.69 (d, *J* = 11.5 Hz, 1H, C*H*HPh), 4.55 (d, *J* = 11.0 Hz, 1H, C*H*HPh), 4.42 (d, *J* = 12.2 Hz, 1H, C*H*HPh), 4.35 (d, *J* = 11.0 Hz, 1H, C*H*HPh), 4.28 (d, *J* = 12.2 Hz, 1H, C*H*HPh), 3.74 (apparent t, *J* = 9.2 Hz, 1H, H-3’), 3.64 (m, 1H, H-5’), 3.59 (dd, *J* = 9.6, 3.6 Hz, 1H, H-2’), 3.53 (apparent t, *J* = 9.6 Hz, 1H, H-4’), 3.37 (m, *2*H, H-6’a, H-6’b); ^13^C NMR (CDCl_3_, 125.8 MHz) δ 170.6 (C-1), 165.7, 165.5, 165.1 (3 × C*O*Ph), 138.6–127.5 (aromatic), 96.6 (C-1’), 81.0 (C-3’), 78.9, 78.8 (C-4, C-2’), 76.9 (C-4’), 75.5 (*C*H_2_Ph), 74.7 (*C*H_2_Ph), 74.2, 74.1 (C-2, C-3), 73.3 (*C*H_2_Ph), 72.4 (*C*H_2_Ph), 71.1 (C-5’), 69.8 (C-5), 67.8 (C-6’), 62.2 (C-6); Anal. calcd for C_61_H_56_O_14_: C, 72.32; H, 5.57; found: C, 72.39; H, 5.65.

**2-*O*-(2,3,4,6-Tetra-*O*-benzyl-α-D-glucopyranosyl)-3,5,6-tri-*O*-benzoyl-α,β-D-galactofuranose (8):** To a solution of bis(2-butyl-3-methyl)borane (4.38 mmol) in anhydrous THF (3.0 mL) cooled to 0 °C under an argon atmosphere, was added a solution of compound **7** (0.44 g, 0.43 mmol) in THF (2.0 mL). The resulting solution was stirred at room temperature for 16 h and then processed as previously described [33]. The organic layer was washed with water, dried (Na_2_SO_4_) and concentrated. Boric acid was eliminated by coevaporations with MeOH. The syrup obtained was purified by column chromatography (hexane/EtOAc/Et_3_N 5:3:0.05). Fractions of *R*_f_ 0.44 (hexane/EtOAc 1:1) afforded syrupy compound **8** (0.32 g, 76%), as a 1:1 α/β anomeric mixture; ^1^H NMR (CDCl_3_, 500 MHz,) only the assigned δ are listed: 5.55 (dd, *J* = 10.0, 4.5 Hz, 0.46H, H-1α), 5.50 (d, *J* = 5.3 Hz, 0.54H, H-1β), 5.27 (d, *J* = 2.0 Hz, 0.54H, H-2β), 5.16 (ddd, *J* = 0.6, 4.3, 7.2 Hz, 0.46H, H-2α), 5.08 (d, *J* = 1.5 Hz, 0.54H, H-1’β), 5.02 (d, *J* = 2.3 Hz, 0.46H, H-1’α), 4.55 (dd, *J* = 5.6, 7.1 Hz, 0.46H, H-3α), 3.76 (d, *J* = 10.0 Hz, 0.46H, HO), 3.02 (d, *J* = 5.3 Hz, 0.54H, HO); ^13^C NMR (CDCl_3_,125.8 MHz) δ 166.2–165.4 (Bz*C*O), 138.7–127.5 (C-aromatic), 100.4, 99.3, 97.2, 96.7, 85.0, 81.9, 81.6, 81.0, 80.9, 79.7, 78.8, 78.7, 77.8, 77.38, 77.33, 77.2, 76.9, 75.5, 74.89, 74.86, 74.2, 73.6, 73.39, 73.37, 73.35, 73.33, 71.3, 70.9, 68.0, 67.9, 62.0, 61.6; Anal. calcd for C_61_H_58_O_14_: C, 72.18; H, 5.76; found: C, 71.93; H, 5.68.

From a faster moving fraction (*R*_f_ 0.68, hexane/EtOAc 1:1), starting compound **7** (0.020 g) was recovered.

**α-D-Glucopyranosyl-2-*O*-α,β-D-galactopyranose (1):** To a solution of compound **8** (0.15 g, 0.14 mmol) in dried CH_2_Cl_2_ (3.0 mL) at 0 °C, 0.1 M NaOMe/MeOH (1 mL) was added. After 1 h the solution was concentrated to 1 mL, deionized by elution with MeOH through a column of strongly acidic cation exchange resin (H^+^). The eluate was concentrated and the syrup obtained (*R*_f_ 0.36, 0.40, 2:1 α and β-anomers, EtOAc) was dissolved in MeOH (1.5 mL) and hydrogenated under 30 psi in the presence of 10% Pd/C. After 24 h the catalyst was filtered over Celite and the filtrate was evaporated under vacuum to afford foamy compound **1** (0.046 g, 91%), *R*_f_ 0.43, *R*_gal_ 0.85 (*n*-PrOH/EtOH/H_2_O 7:1:2), [α]_D_ +130 (*c* 1, H_2_O) (lit. [21]: [α]_D_ +142 (*c* 0.6, H_2_O)); ^1^H NMR (D_2_O, 500 MHz) anomeric region for the major isomer α-D-Glc*p*(1→2)-D-Gal*p*: δ 5.46 (d, *J* = 3.6 Hz, 1H, H-1α), 5.37 (d, *J* = 3.9 Hz, 1H, H-1’α), 5.10 (d, *J* = 3.8 Hz, 1H, H-1’β), 4.73 (d, *J* = 7.8 Hz, 1H, H-1β); ^13^C NMR (D_2_O, 125.8 MHz) δ 97.9 (C-1’ β-anomer), 96.6 (C-1 β-anomer), 96.1 (C-1’ α-anomer), 89.6 (C-1 α-anomer), 76.9, 75.0, 73.1, 72.7, 71.8, 71.6, 71.4, 71.3, 70.4, 69.46, 69.42, 69.0, 67.5, 61.0, 60.9, 60.4, 60.3; HRMS–ESI(APCI, *m*/*z*): [M + NH_4_]^+^ calcd for C_12_H_22_O_11_, 360.1506; found, 360.1518.

### Isolation of varianose

*Penicillium varians* (CBS 386.48) was purchased from Centraalbureau voor Schimmelcultures, Baarn-Delft (the Netherlands). The culture was maintained according to Haworth et al. [1], and grown under the conditions previously described, with glucose (5 g/L) as a carbon source according to Bordoni et al. [17].

Mycelium was separated from the supernatant by centrifugation (10.000 rpm, 5 °C, 20 min). Culture supernatant was concentrated by lyophilization to a final volume of 50 mL, and the polysaccharide varianose was precipitated by addition of 200 mL ethanol and left overnight at 5 °C. The pellet (V_1_) was separated and the process repeated once more, after concentration of the supernatant to 50 mL. A new pellet (V_2_) was obtained by centrifugation. Both V_1_ and V_2_ fractions were pooled, resuspended in water, and lyophilized to obtain a white solid (varianose).

### Structural characterization of varianose and isolation of α-D-Glc*p*(1→2)-D-Gal (**1**)

In order to obtain the disaccharide α-D-Glc*p*(1→2)-D-Gal (**1**), a varianose sample (10 mg/mL) was hydrolyzed with 0.1 M TFA for 90 min h at 85 °C. The solution was then concentrated by using a rotary evaporator, and the trifluoracetic acid was coevaporated with water to neutral pH. The residue was chromatographed on a Biogel P-2 column and eluted with water collecting 1 mL fractions (V_o_ 65 mL, V_i_ 137 mL), which were measured for neutral carbohydrate content by the phenol–H_2_SO_4_ method (200 µL aliquots).

Aliquots of the Biogel P-2 included fraction, containing the natural disaccharide, were analysed by HPAEC–PAD (condition 1). A sample of this fraction was hydrolyzed with TFA 2 N, 105 °C, 3 h for total monosaccharide composition analysis by HPAEC–PAD (condition 2).

### HPAEC–PAD analysis

Analysis by HPAEC–PAD was performed by using a Dionex ICS-3000 HPLC system equipped with a pulse amperometric detector (PAD). The detector was set at 30 nA and E1 = +10.05 V, E2 = +10.60 V, and E3 = −0.60 V. The column used was a CarboPac PA-10 anion-exchange analytical column (4 × 250 mm), equipped with a guard column PA-10 (5 × 50 mm). The following conditions were used: **Condition 1** (disaccharides): 100 mM NaOH, isocratically at a flow rate of 1 mL/min. **Condition 2** (monosaccharides): 18 mm NaOH, isocratically, at a flow rate of 1 mL/min.

## Supporting Information

File 1 NMR spectra of compounds **1**, **5**, **7** and **8**.
