# Supplementary material for: Synthesis of a derivative of α-D-Glcp(1->2)-D-Galf suitable for further glycosylation and of α-D-Glcp(1->2)-D-Gal, a disaccharide fragment obtained from varianose
Source: Beilstein J Org Chem. 2012 Dec 7;8:2142–8. doi: 10.3762/bjoc.8.241 (PMC3520571; doi:10.3762/bjoc.8.241)
Supplement: File 1 — NMR spectra of compounds 1, 5, 7 and 8. [file Beilstein_J_Org_Chem-08-2142-s001.pdf]

Supporting Information

for

Synthesis of a derivative of α-D-Glc*p*(1→2)-D-Gal*f* suitable for further glycosylation and of α-D-Glc*p*(1→2)-D-Gal, a disaccharide fragment obtained from varianose

Carla Marino[1]*, Carlos Lima[1], Karina Mariño[1,2] and Rosa M. de Lederkremer[1]

Address: [1]CIHIDECAR-CONICET-UBA, Departamento de Química Orgánica, Facultad de Ciencias Exactas y Naturales, UBA, Buenos Aires (1428), Argentina and [2]Laboratorio de Glicómica Funcional y Molecular, Instituto de Biología y Medicina Experimental (IBYME), CONICET, Buenos Aires (1428), Argentina

Email: Carla Marino* - cmarino@qo.fcen.uba.ar

*Corresponding author

NMR spectra of compounds 1, 5, 7 and 8

Table of Contents

^{1}H NMR spectrum of compound **5** S2

^{13}C NMR spectrum of compound **5** S3

^{1}H NMR spectrum of compound **7** S4

^{13}C NMR spectrum of compound **7** S5

^{1}H NMR spectrum of compound **8** S6

^{13}C NMR spectrum of compound **8** S7

^{1}H NMR spectrum of compound **1** S8

^{13}C NMR spectrum of compound **1** S9

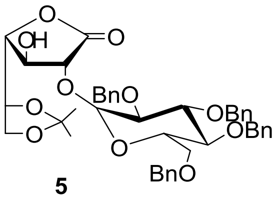

5

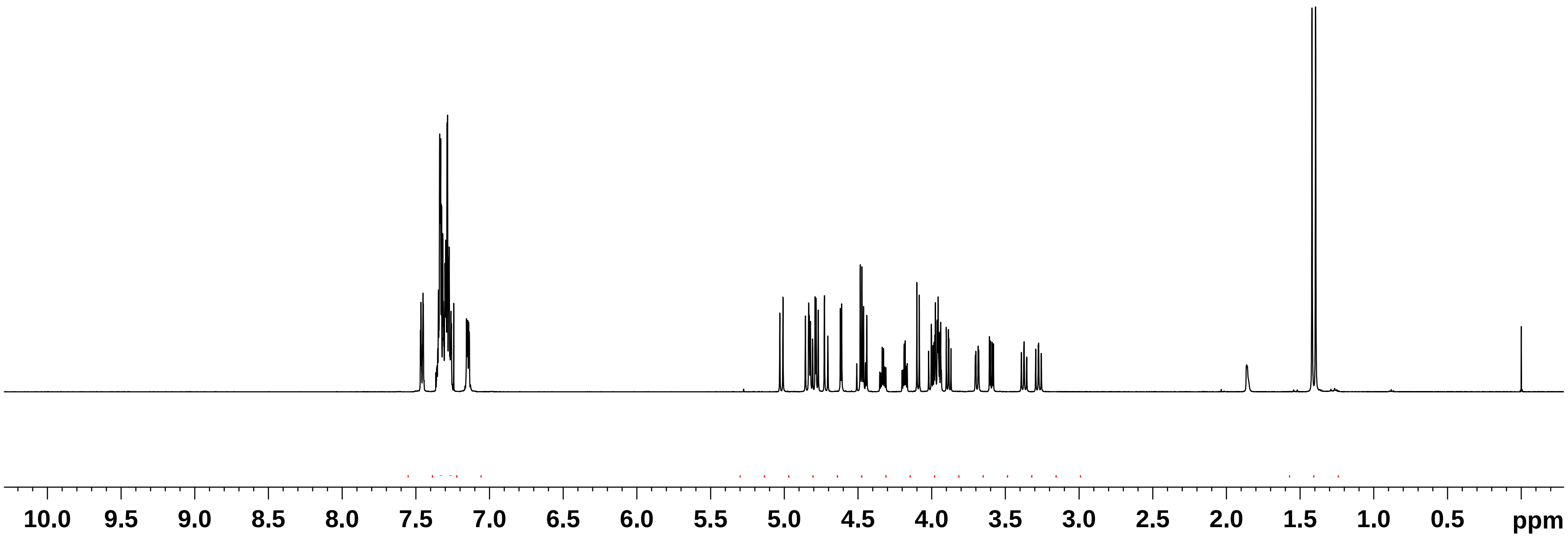

Figure S1: ^{1}H NMR of compound **5**.

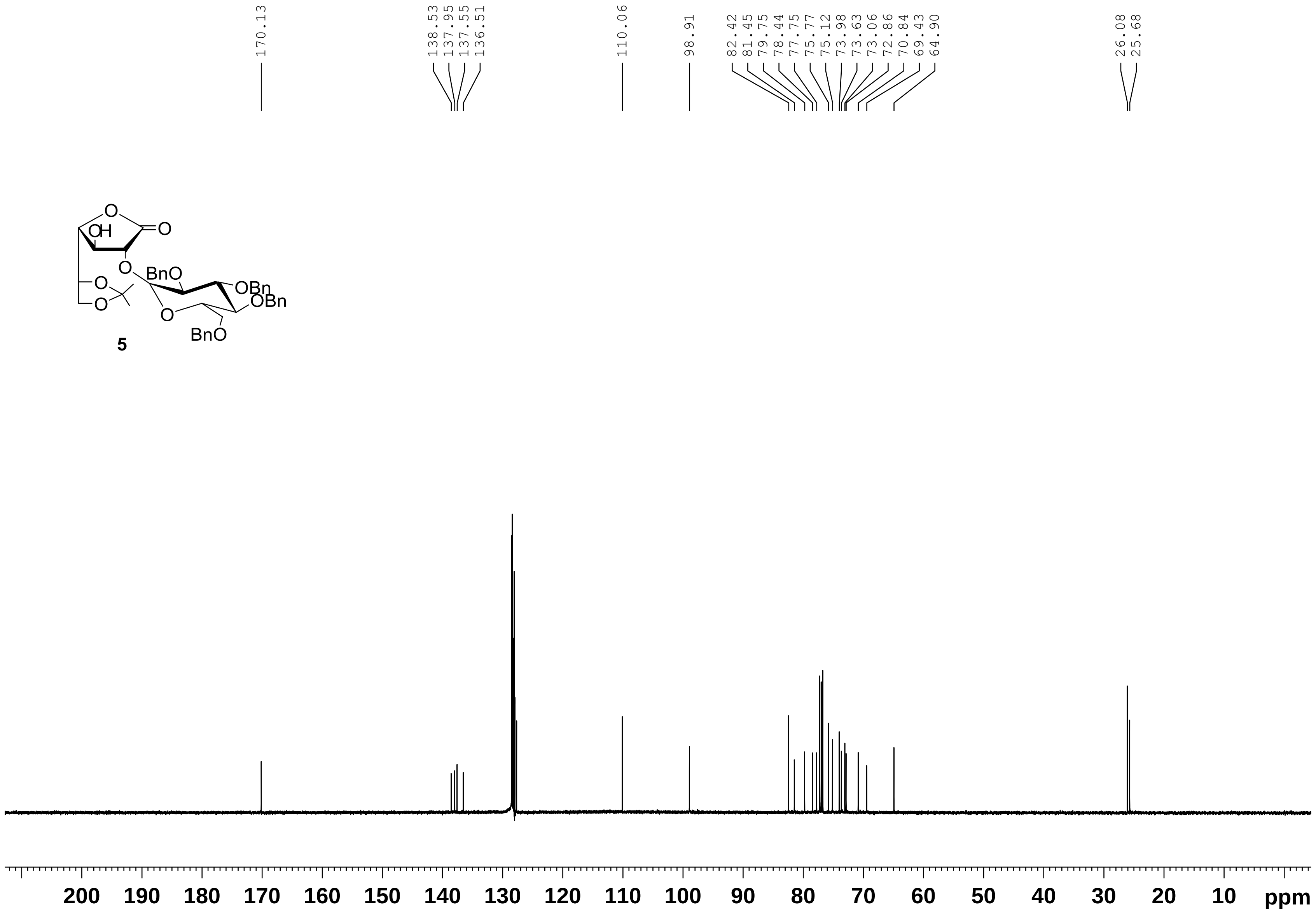

Figure S2: ^{13}C NMR of compound **5**.

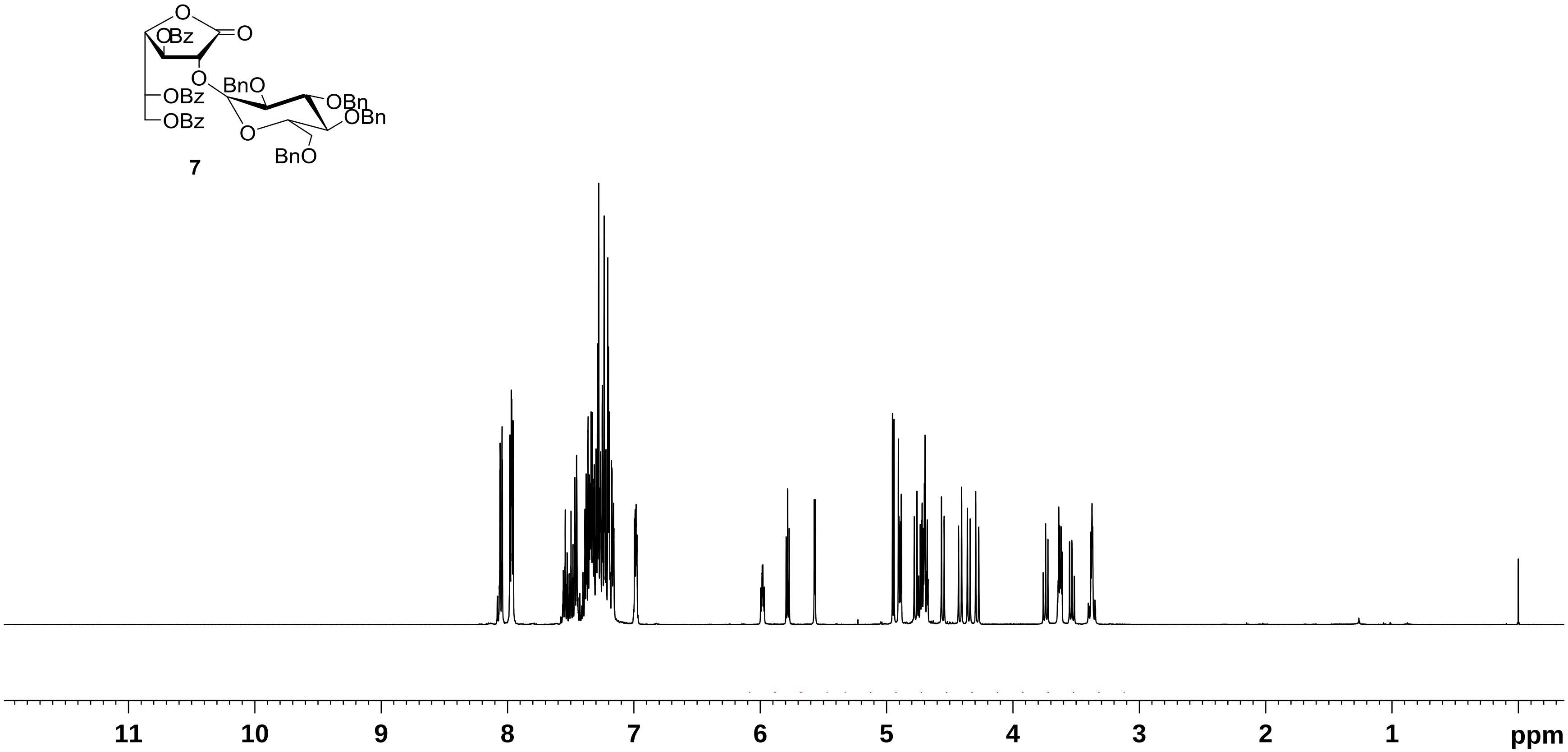

Figure S3: ^{1}H NMR of compound **7**.

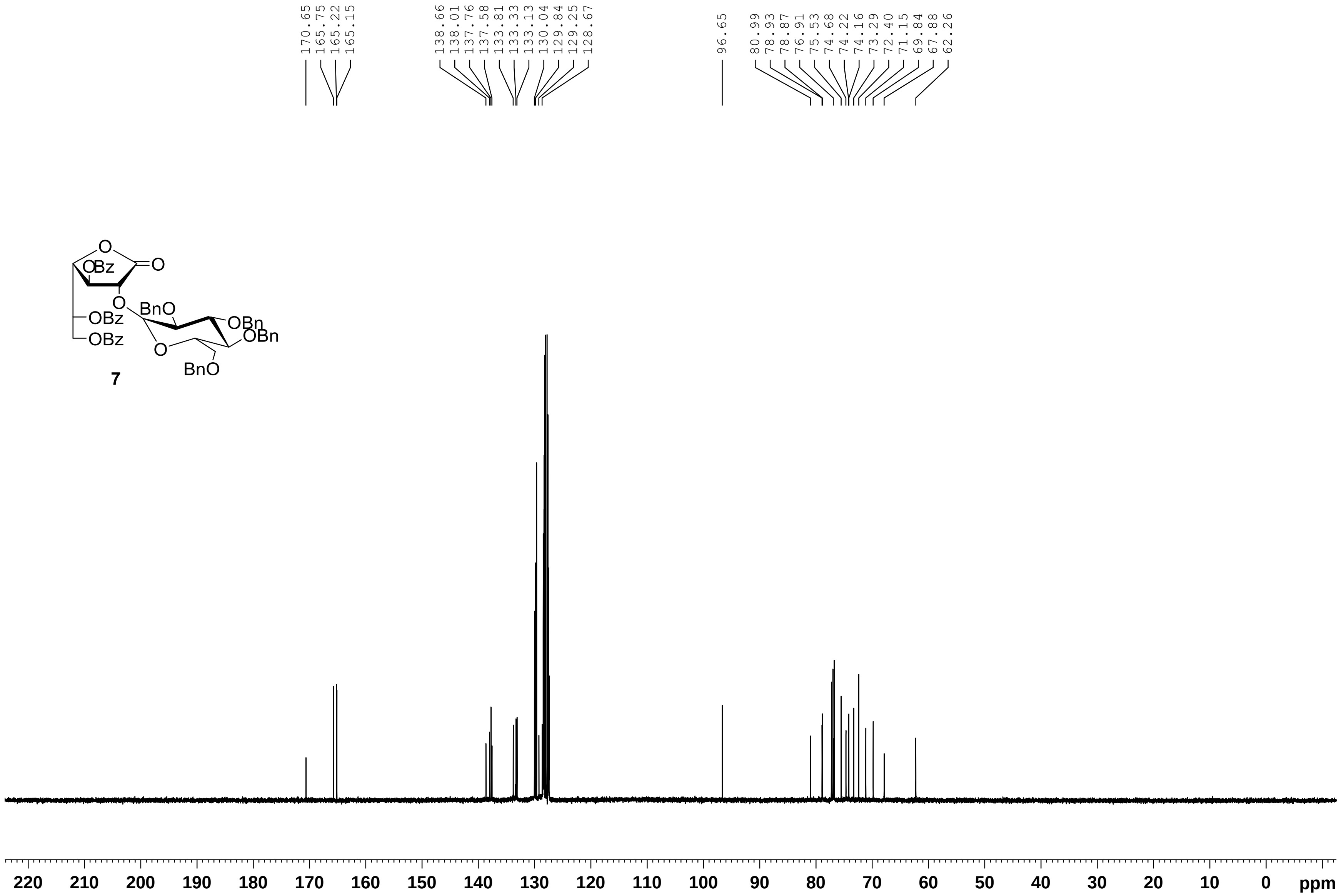

Figure S4: ^{13}C NMR of compound **7**.

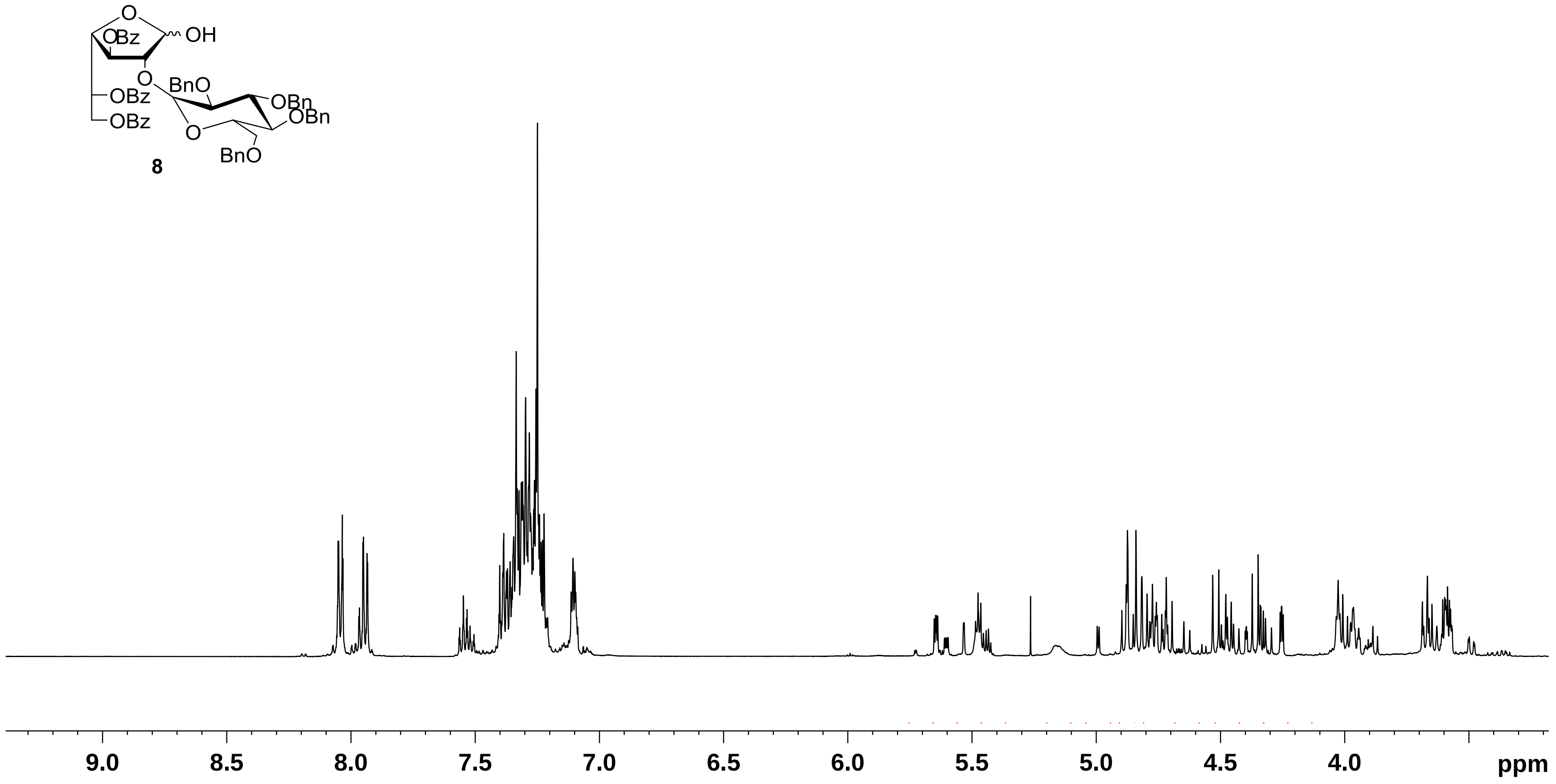

Figure S5: ^{1}H NMR of compound **8**.

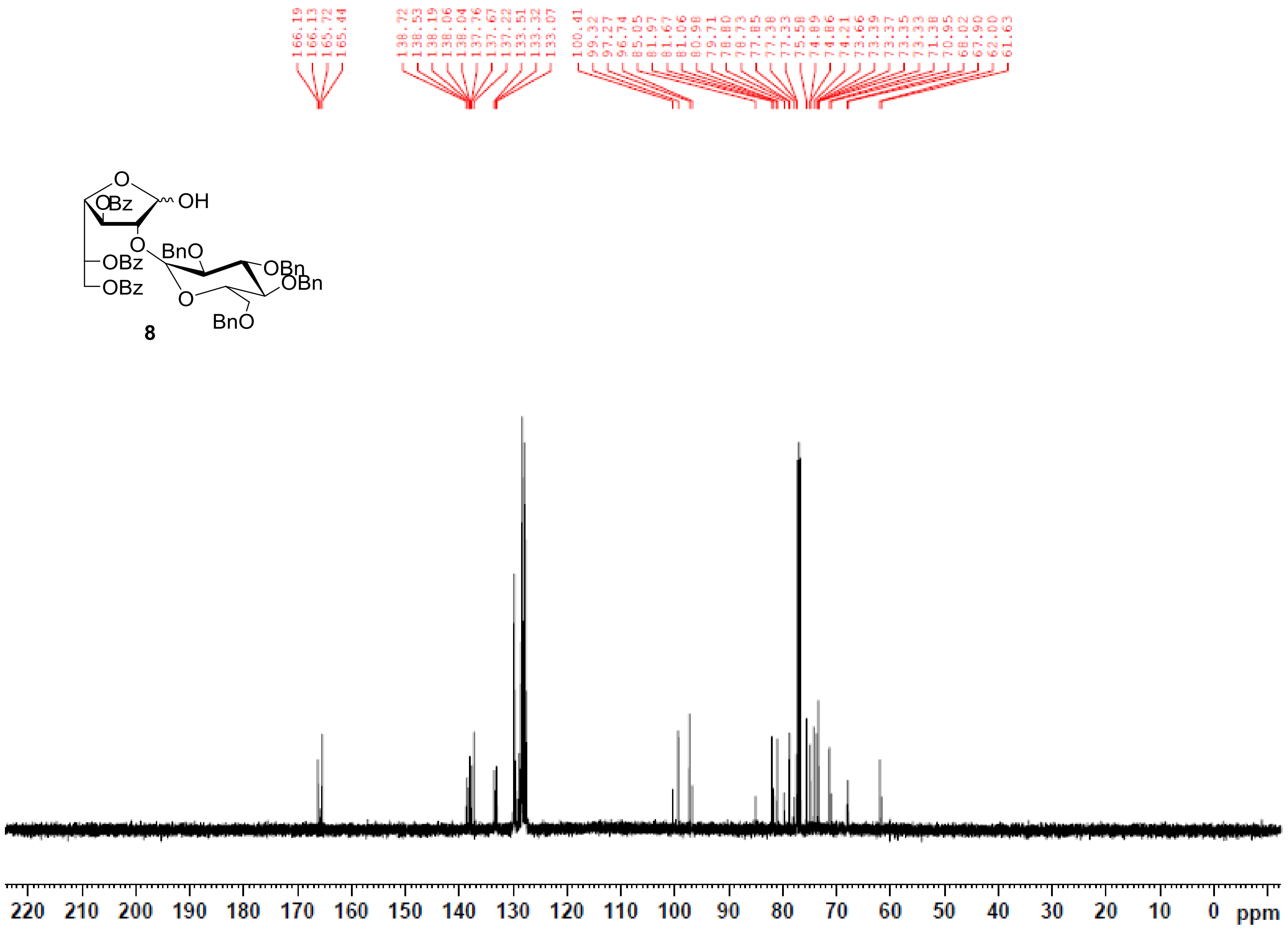

Figure S6: ^{13}C NMR of compound **8**.

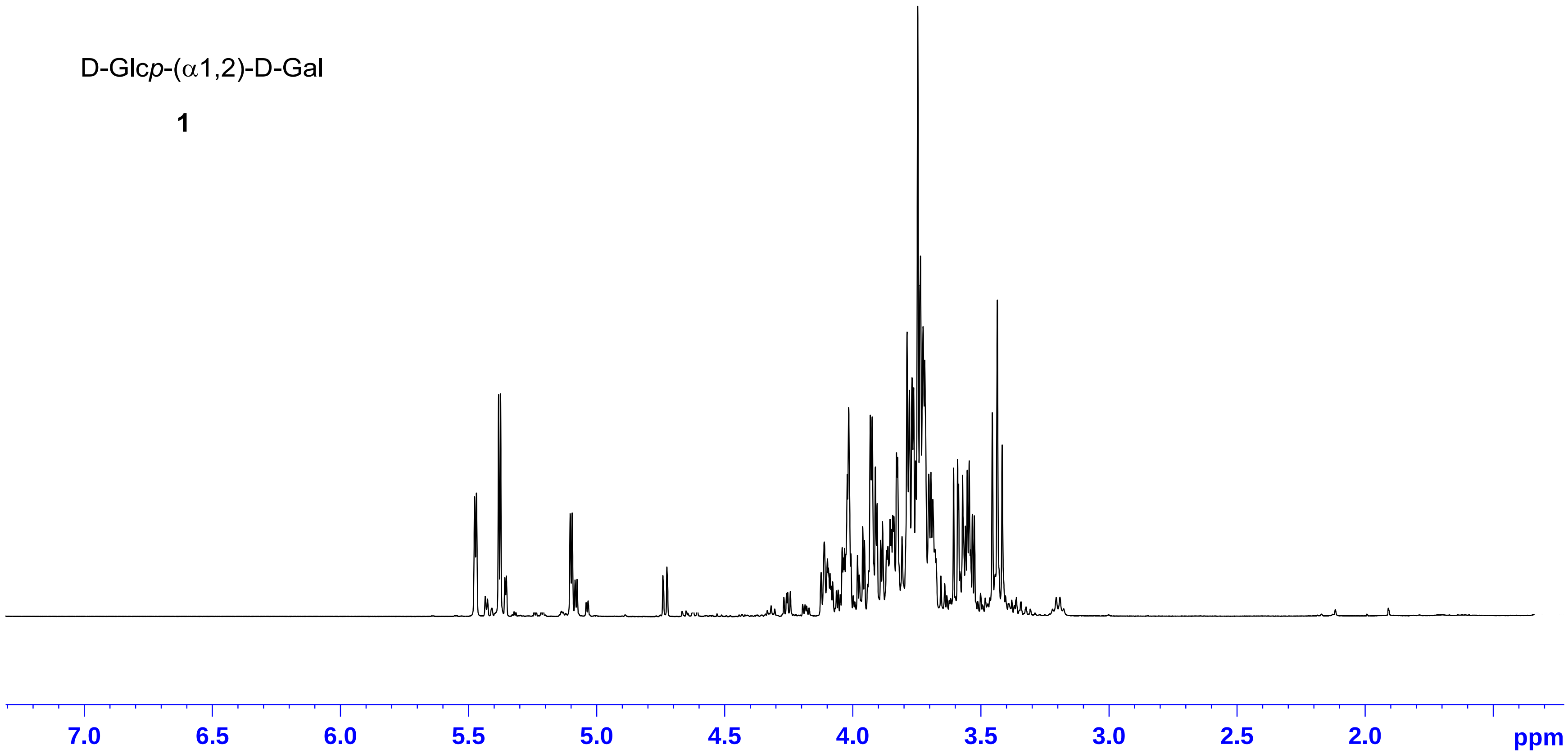

Figure S7: ^{1}H NMR of compound **1**.

D-Glc*p*-(α1,2)-D-Gal

1

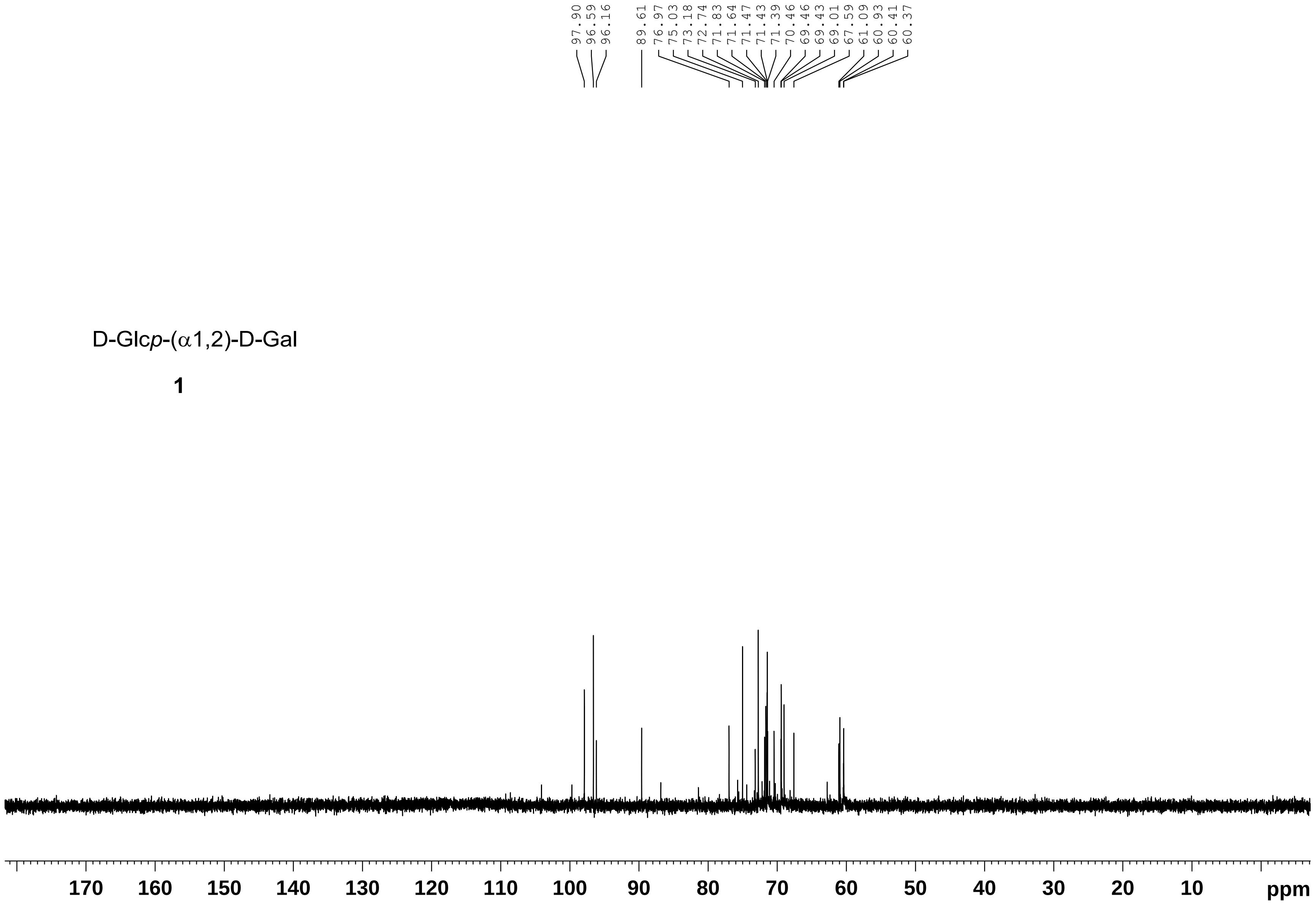

Figure S8: ^{13}C NMR of compound **1**.
